# The Role of Matrix Metalloproteinases in Diabetic Wound Healing in relation to Photobiomodulation

**DOI:** 10.1155/2016/2897656

**Published:** 2016-05-23

**Authors:** Sandra Matabi Ayuk, Heidi Abrahamse, Nicolette Nadene Houreld

**Affiliations:** Laser Research Centre, Faculty of Health Sciences, University of Johannesburg, P.O. Box 17011, Doornfontein 2028, South Africa

## Abstract

The integration of several cellular responses initiates the process of wound healing. Matrix Metalloproteinases (MMPs) play an integral role in wound healing. Their main function is degradation, by removal of damaged extracellular matrix (ECM) during the inflammatory phase, breakdown of the capillary basement membrane for angiogenesis and cell migration during the proliferation phase, and contraction and remodelling of tissue in the remodelling phase. For effective healing to occur, all wounds require a certain amount of these enzymes, which on the contrary could be very damaging at high concentrations causing excessive degradation and impaired wound healing. The imbalance in MMPs may increase the chronicity of a wound, a familiar problem seen in diabetic patients. The association of diabetes with impaired wound healing and other vascular complications is a serious public health issue. These may eventually lead to chronic foot ulcers and amputation. Low intensity laser irradiation (LILI) or photobiomodulation (PBM) is known to stimulate several wound healing processes; however, its role in matrix proteins and diabetic wound healing has not been fully investigated. This review focuses on the role of MMPs in diabetic wound healing and their interaction in PBM.

## 1. Introduction

Wound healing is a highly coordinated and carefully orchestrated process to promote proper healing [1, 2]. This process forms the ECM that enhances cellular processes in the wound, including cell adhesion, migration, and tissue remodelling. Matrix Metalloproteinases (MMPs) are zinc endopeptidases capable of degrading all components of the extracellular matrix (ECM) [3]. They participate in all the phases of wound healing by removing damaged proteins and temporary ECM during the inflammatory phase, breaking down the capillary basement membrane in the proliferation phase for angiogenesis and cell migration, as well as contracting and remodelling tissue in the remodelling phase. In addition, MMPs interplay the activity of a few growth factors and polymorphonuclear cells (PMNs) [4–10].

The MMP family comprises enzymes based on their substrate specificity, collagenases (MMP-1, MMP-8, MMP-13, and MMP-18), gelatinases (MMP-2 and MMP-9), stromelysins (MMP-3, MMP-10, and MMP-11), membrane type (MT) metalloproteinases (MMP-14, MMP-15, MMP-16, MMP-17, MMP-24, and MMP-25), matrilysins (MMP-7 and MMP-26), and others (MMP-12, MMP-19, MMP-20, MMP-21, MMP-23, MMP-27, and MMP-28) [11, 12]. They are comprised of three histidines conserved on the zinc site as well as a conserved methionine below the active site. Gelatinases (MMP-2 and MMP-9) and collagenases (MMP-1 and MMP-8) are particularly involved in the wound healing process. Tissue inhibitor of metalloproteinase (TIMP), of which there are four different subtypes (TIMP-1, TIMP-2, TIMP-3, and TIMP-4), interrupts the action of MMPs and thus regulates their action. TIMPs attach to alternate or active sites of MMPs and are capable of inhibiting all MMPs, although their affinity levels may differ [13]. Even though the importance of MMPs in wound healing cannot be underestimated, they can negatively affect healing if not present in the correct amount [14, 15]. Under normal conditions, the interaction of MMPs with growth factors and receptors is capable of maintaining the balance of the ECM. In such circumstances, the amount of MMP is decreased. However, predictors of healing will depend on the ratio between MMPs and TIMPs [16].

Within the ECM, an imbalance of these matrix proteins and their inhibitors may cause degradation of the matrix [17] and is associated with proteolytic enzymes distributed in several groups involved in disease development [18]. The interaction lowers growth factor and TIMP levels. It also increases the level of proteases and MMPs, especially MMP-1, MMP-2, MMP-8, and MMP-9, and proinflammatory cytokines such as interleukin-1 (IL-1) and tumour necrosis factor-alpha (TNF-*α*) released by neutrophils and macrophages. The effect is higher in chronic wounds as opposed to acute wounds. The imbalance is associated with abnormal wound healing conditions such as those in diabetes mellitus (DM) and other vascular complications [19, 20].

## 2. Mechanism of Matrix Metalloproteinases in Delayed Wound Healing

MMPs control the composition of ECM in various vascular and nonvascular inflammatory conditions [4, 5, 7–9, 21]. The ECM supports the vascular walls through the action of MMPs and their inhibitors. When an injury occurs, in the vascular system, it initiates an inflammatory reaction. The interaction of MMPs in disease pathogenesis is very critical. Some of the events include inflammation, activation of PMNs, and release of MMPs. Inflammatory cytokines such as IL1-*α*, IL1-*β*, IL-2, IL-17, C-reactive protein (CRP), insulin-like growth factor-1 (ILGF-1), and TGF-*α* stimulate the production of neutrophil gelatinase-associated lipocalin (NGAL). NGAL then activates MMP activities especially MMP-9 to form MMP-9/NGAL complex. This process occurs during the onset and healing phases of nonhealing venous ulcers [9, 22, 23]. This serves as a contributing factor to delayed wound healing even though it is not clear how many biochemical factors are involved.

Several studies have supported the central role played by NGAL in the activation of MMPs, especially MMP-9 in delayed wound healing [7–10, 22, 24]. Amato et al. [7] observed that MMP-1 and MMP-8 are pivotal in normal wound healing; however, overexpression was seen in patients with nonhealing chronic venous ulcers (CVUs) as compared to patients with healing ulcers undergoing normal and physical treatment. Similarly, Serra et al. [8] also found MMP and NGAL to be pivotal in arterial aneurysms due to high levels seen in patient's plasma. They concluded that the use of MMPs as molecular markers to prevent aneurysmal rupture was vital. In contrast, Karlsson et al. [25] did not find any correlation between MMP, NGAL, and inflammatory cytokines in their investigation. Most authors concluded and linked the association of MMP/NGAL to the pathophysiology of chronic wounds during the inflammatory and proliferative phases, whereby proteases appear to be significantly high, resulting in repeated migration of neutrophils, PMNs, and macrophages to the wound site. They concluded that overexpression of these proteins could have prolonged the inflammatory phase due to excessive collagenolytic properties and neutrophils resulting in delayed wound healing [8–10, 22, 24].

Furthermore, the production of MMPs and endotoxins, provoked by high metabolic activity of bacteria due to replication, has influenced the wound progression at different stages negatively. Infections are predominant with improper regulation of the immune defence system and a factor favouring bacterial growth, which may cause the wound to remain chronic, except for the eradication of the causative agent. Many studies have confirmed the major role of MMPs in infectious diseases [10, 26–28]. During unfavourable conditions, MMP degrades the ECM causing immune cells to migrate to initiate inflammatory response or proteolysis to eradicate the pathogen [29, 30]. The bacterial colonisation depends on several factors and patients with persistent infections are more predisposed to prolonged healing and care [31, 32].

## 3. Interaction of Matrix Metalloproteinases (MMPs) in Diabetic Wound Healing

**Figure 1:**
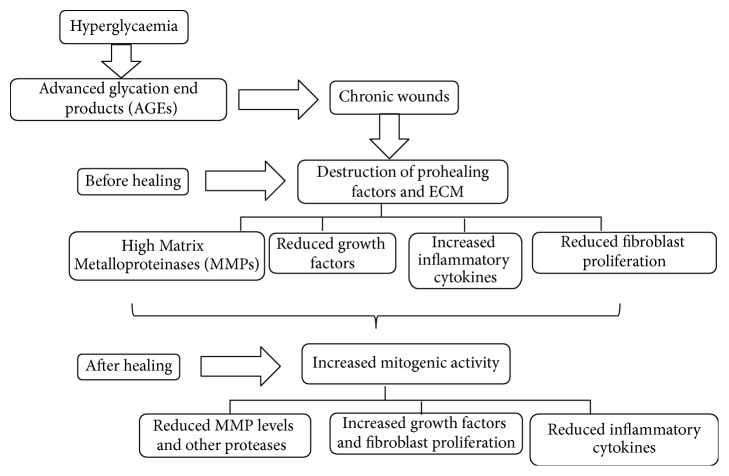
Mechanism of MMPs in chronic wounds. The development of chronic wounds results from delayed inflammation producing high levels of proteinases that destroy the essential elements of wound healing, growth factors, various receptors, and ECM proteins leading to increased MMPs, inflammatory cytokines, and reduced growth factors. The destruction of prohealing factors and ECM also reduces the ability for cells to migrate and proliferate. However as the wound begins to heal through various therapies, there would be increased mitogenic activity, reduced protease activity, and reduction of inflammatory cytokines.

Diabetes is a chronic metabolic disorder affecting millions of people globally [33]. Hyperglycaemia induces the majority of the micro- and macrovascular complications associated with DM [20] and increases MMP activity directly or indirectly through oxidative stress or advanced glycation end products (AGEs) (Figure 1) [14, 20, 34]. There are significantly higher levels of MMP in patients with metabolic syndrome as compared with normal individuals. This indicates that such patients may have high tendencies of developing other physiological problems [13]. The process of wound healing necessitates ECM degradation to be controlled; thus an imbalance between ECM formation and the degradation process could lead to the development of chronic ulcers or fibrosis [35]. Cellular and biochemical imbalances, tissue damage, or other disease conditions may present varied effects in the healing process. This also upsets the proteases, cytokines, and growth factors leading to an absence or delay of wound closure preventing successful skin repair [14, 15, 36]. There is a serious concern in the health sector regarding the unresolved problems associated with venous leg ulcers (VLU) and diabetes, which may not only be associated with pain, depreciated life, and/or eventual death, but also pose a severe economic burden [37–39]. Diabetic foot ulcer is common among any age group [40] and is predominantly seen in those over the age of 65 years with an occurrence of more than 3% [41] and an incidence of more than 1% of the world population [42].

Enzyme activity affected by hyperglycaemia disrupts the expression of MMPs in diabetes. An increased proteolytic environment provoked by an alteration in MMPs and TIMPs affects patients with diabetic ulcers [43]. Recent indications have shown the association of MMPs and their inhibitors with diabetic development and progression to be poor [43, 44]. Increased activities and levels of MMP-1, MMP-8, and MMP-9 have been identified in slow-to-heal wounds, with relatively low TIMP levels. Neutrophil derived MMP-8 may be up to 50–100 times higher as compared to normal wounds due to chronic inflammation [15, 45–47]. MMP-9 degrades fibronectin into fragments, which further activates MMP, cell migration, and proliferation. This provokes white blood cell infiltration, tissue damage, and continuous inflammation [48]. MMP-1, MMP-8, and MMP-9 are highly expressed in venous wounds in the absence of TIMPs [5, 7, 23]. Pirilä et al. [49] studied the expression of MMP-8 and MMP-26 in human cutaneous wounds and concluded that MMP-8 and MMP-26 could be associated with both acute and chronic wounds. Khattri and colleagues [13], after examining 388 subjects, found an overexpression of MMP-2 and MMP-9 in the serum of patients with metabolic syndrome as compared to controls. They concluded that altered expression of MMPs may provoke pathogenesis in several tissues [13, 50, 51]. Singh et al. [52] elucidated altered gene expression in MMP-9 as a cause of nonhealing diabetic ulcers, whereby diabetic wounds as compared to the controls had significant unmethylated MMP-9 gene promoter status that could be a result of impaired MMP expression in such wounds. According to Lobmann et al. [53], MMP-1, MMP-2, MMP-8, and MMP-9 were highly expressed in normal and chronic diabetic wounds with a decrease in TIMP-2. They concluded that this could be due to high proteolytic surroundings promoting poor healing in diabetes. Similarly, there was an overexpression of MMP-1 and MMP-9, as well as TIMP-1, in keratinocytes derived from foot ulcers in diabetic type 1 patients. This study supports the upregulation of certain MMPs and TIMPs in diabetic foot ulcers [54]. In addition, Menghini and colleagues [43] found MMP-9 to be increased and TIMP-3 to be decreased in diabetic type 2 patients. They suggested that the progression of ulcers could be due to a poor proteolytic environment. They concluded that new therapeutic strategies should focus on increasing the expression levels of TIMP-3. Dinh and colleagues [55] found increased expression of MMP-9, TNF-*α*, and other growth factors in diabetic foot ulcers and concluded that they could be linked with slow-to-heal ulcers in diabetics and therefore a target for new therapeutic management. These studies have all ascertained that MMPs and TIMPs are elevated in chronic wounds; however, they may also play a role in determining the level of chronicity [13]. Uccioli et al. [56] elucidated that chronicity is associated with an increase mainly in MMP-9 and MMP-8 and elastase activity that may eventually alter collagen synthesis and the release of growth factor and cytokines into the site of injury.

An increased activity of MMPs may initiate the development of diabetic peripheral arterial disease. Death and colleagues [57] found that hyperglycaemia affected the regulation of MMP/TIMP and increased the activities of MMP-1, MMP-2, and MMP-9 in vascular cells, stimulating the degradation of the ECM and causing an imbalance in diabetes. Chung and colleagues [34] elucidated that an increase in expression of MMP-2 and MMP-9 as well as protein expression of TIMP-1 may be a resulting factor in impaired wound healing and might provide an explanation for human arterial vasculature in type 2 DM.

## 4. Matrix Metalloproteinases (MMPs) and Current Therapeutic Strategies

Some of the pathophysiological events responsible for initiating CVUs are inflammation, PMN activation, and the release of proteases, especially MMPs. Many different compounds incorporated into conventional treatment have improved the quality of life in patients suffering from difficult-to-heal wounds. Compounds such as bioflavonoids, nutraceuticals, and glycoaminoglycans such as sulodexide (SDX) and cilostazol, together with conventional treatments, have improved healing and clinical conditions of patients [4, 5, 10]. In a recent study done by Serra and colleagues [10] on the effects of new nutraceutical substances, the researchers found that there was an overexpression of MMPs, NGAL, and inflammatory cytokines in CVUs on treating patients conventionally. They introduced a nutraceutical substance together with compression therapy and surgical correction for 8 months and found a decrease in inflammatory cytokines with improved wound healing. Their findings explained that there is a correlation between the pathophysiology and impaired wound healing probably due to the overexpression of MMPs, NGAL, and inflammatory cytokines. Their findings are consistent with other studies using glycoaminoglycans such as sulodexide (SDX) and cilostazol [4, 5]. The authors concluded that inhibition could be a possible therapeutic intervention. In addition to these compounds, the use of biomarkers would be vital when clinical signs are misleading [58, 59]. However, mimicking the action of MMP inhibitors could possibly assist in managing infected wounds or wounds activated through MMP or cytokine release [4, 5, 10, 60].

## 5. Matrix Metalloproteinases (MMPs), Wound Healing, and Photobiomodulation (PBM)

**Figure 2:**
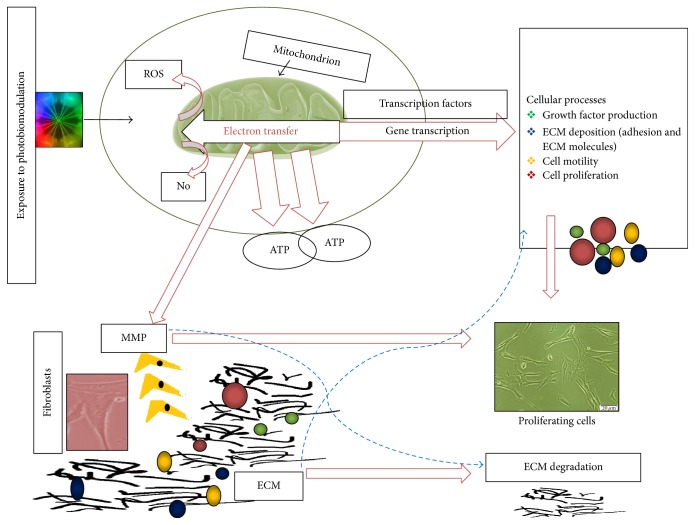
The interaction of photobiomodulation (PBM) and the extracellular matrix (ECM). Light absorbed by photons in the electron transport chain within the mitochondria generates the production of Adenosine Triphosphate (ATP), Reactive Oxygen Species (ROS), and Nitric Oxide (NO). This evokes the release of transcription factors leading to the transcription of genes, which develop and enhance various cellular processes. Matrix Metalloproteinases (MMPs) are associated with changes that occur in the ECM. Under disease conditions, excess MMP is expressed which leads to the enhanced degradation of the ECM. When cells are exposed to PBM, there is an enhancement in the release of various cells including fibroblasts, growth factors, and adhesion molecules into the basement membrane leading to ECM remodelling.

PBM is a noninvasive form of light therapy for wound healing, whereby several biological, chemical, and cellular processes are stimulated to speed up healing (Figure 2). The adequacy of this therapy is specific to a spectral range of 500–1,100 nm and power output ranging within 10–200 mW [61]. Investigations carried out found PBM to enhance wound healing [62–64]. Studies have also established its effect on wounded cell models* in vitro* using various wavelengths and dosages [65–67]. In addition, studies performed on mice, rats, and humans showed a stimulatory effect of PBM in diabetic wound healing [62, 63, 68–71].

The application of PBM in altering the gene expression in cell cultures is evident [72, 73]. PBM is able to alter the expression of MMPs in diabetic wounds and enhance collagen production [64, 74, 75]. However, experiments done on human skin fibroblasts demonstrated variations in gene expression [75, 76]. Ayuk and colleagues [75] found that PBM altered 49 genes involved in the ECM* in vitro*, with genes in a diabetic wounded cell model mostly downregulated, among which were MMP-1, MMP-2, MMP-8, MMP-12, MMP-14, and MMP-16. PBM in another study (1 or 3 J/cm^2^ with 810 nm) was shown to reduce the gene expression of MMP-3, MMP-9, and MMP-13 in Achilles tendons of rats as well as prostaglandin, implying that it was able to reduce tendon inflammation and preserve elasticity and resistance [77]. Casalechi and colleagues [78] investigated a nonconventional treatment for tendinopathies and found that PBM (780 nm; 107 mW/cm^2^; 7.5 J/cm^2^) alongside various inflammatory treatments modulated mRNA gene expression of MMP-1, MMP-13, and IL-10 and vascular endothelial growth factor (VGEF) in a Wistar rat model. Cury et al. [79] conducted an experiment using sixty male Wistar rats which were irradiated at 660 and 780 nm with a fluence of 30 and 40 J/cm^2^ and found a decrease in MMP-2 activity and increased expression in angiogenesis markers such as VEGF. Therefore, PBM was associated with promoting healing of skin flaps in a dose dependent manner. PBM also plays a role in regulating inflammatory cytokines. In a study conducted by Laraia and colleagues [80] to analyze the role of PBM (660 nm; 100 mW) on protein expression levels in rat Achilles tendons with their main focus on the inflammatory mediators, they found that the group treated with lasers showed positive responses on IL-6 and IL-10 after 6, 24, and 72 h compared to the control. They concluded that PBM plays a significant role in regulating inflammatory cytokines after injury.

Ye et al. [81] used three different dosages (0, 0.6, 1.5, and 2.5 J/cm^2^) at 1064 nm on the dorsal skin of Sprague-Dawley rats and showed an increase in collagen content, as well as the upregulation of the gene expression of collagen type 1 (Col-1) and collagen type 3 (Col-3) and TIMP-1 and TIMP-2, and a significant decrease in MMP-2 and MMP-3. They concluded in their study that irradiation at 1064 nm could markedly increase collagen production and prevent degradation and activate the Erk1/2 and JNK-MAPK pathway. In addition, Lee et al. [82] found that long-pulsed Nd:YAG laser (1064 nm) decreased the expression of MMP-1 at low energy dosages; meanwhile there was an increase in dermal collagen and transforming growth factor-beta (TGF-*β*) with no significant difference in the expression of TIMP-1 in hairless mice. A similar study using cultured human skin fibroblasts after two successive irradiations with 1.5 J/cm^2^ at wavelengths of 1064 and 532 nm significantly increased Col-1 and Col-3 at 24 and 48 h after irradiation. This study also found an increase in the expression of TIMP-1 and TIMP-2 and a decrease in MMP-1 and MMP-2. It ascertained that 1064 nm was more effective as compared to 532 nm in promoting molecular activities [83]. Kim et al. [84] found PBM (780 nm; 1.75 W/cm^2^; 5 J/cm^2^) to modulate the gene expression of five MMPs (MMP-1, MMP-2, MMP-8, MMP-9, and MMP-13) in the periodontal ligament of Sprague-Dawley rats and inhibited immunoreactivity of TIMP-1. Similarly, PBM (780 nm; 125 mW/cm^2^; 0, 0.5, 1.0, 2.5, 5.0, and 20 J/cm^2^) with different energy densities modulated the gene expression of MMP-2 and MMP-9 as well as the oxidative metabolism of the masseter muscle in rats, indicating possible ECM remodelling [85].

The biostimulatory effect of PBM in the near infrared (NIR) range modulates wound healing events in various cells. Gavish et al. [86] found that PBM (780 nm, 1 or 2 J/cm^2^) increased collagen activity twofold, increased MMP-2 activity, upregulated MMP-1 and TIMP-2 expression, and downregulated MMP-2 and IL-1*β*. Their findings may be of therapeutic importance in situations with depleted smooth cells, weakened ECM, and increased proinflammatory markers as major pathological components. Migliario et al. [87] observed a decrease in MMP-9 in normal keratinocytes exposed to laser irradiation at 635.2 nm and a fluence of 82.5 or 112.4 J/cm^2^, a very high dosage, which could influence the production of enzymes during wound healing and probably explain the decrease in cell proliferation. In addition, irradiation using a diode laser prototype (780 ± 3 nm, 40 mW) at different doses of 0.5, 1.5, 3.5, and 7 J/cm^2^ over 24 h showed that a dose range of 0.5–3 J/cm^2^ promotes a biostimulatory effect on keratinocytes [88]. Luo and colleagues [89] found that intense pulse light (IPL) irradiation had a time dependent effect on mouse skin at 2 weeks up to 8 weeks with MMP-1 and MMP-2 showing a decrease in mRNA expression after irradiation, and the authors concluded that irradiation with IPL modulated collagen synthesis as well as ECM degradation.

After treating patients with laser therapy (660 nm; power output 25 mW; dose 6.2 J/cm^2^), Oton-Leite and colleagues [90] found that there was a trend in the reduction levels of IL-1*β*, IL-10, TNF-*α*, TGF-*β*, MMP-2/TIMP-2, and MMP-9/TIMP-2 as compared to the untreated group, even though there was no significant difference. They concluded that it was effective in reducing pain, inflammation, and repair. Silva and colleagues [91] performed a randomized preliminary study to evaluate PBM using saliva and blood from haematopoietic stem cell transplantation patients and found a positive response in MMP-2 in the saliva and IL-10 in the blood. They found out that PBM is clinically effective in reducing chemotherapy induced oral mucositis; however, there was no mechanism linked with inflammatory cytokines, growth factors, and MMPs. Furthermore, some researchers performed a controlled randomized clinical trial and found that after treatment for chronic periodontitis there was a significant difference in the levels of MMP-1, MMP-8, and TIMP-1 between the various groups as compared with the controls. They found a significant decrease in the total amount of IL-1*β*, IL-6, MMP-1, MMP-8, and TIMP-1 with MMP-8 significantly affected. The researchers concluded that MMP-8 has the most effective impact for use as an adjunct laser treatment on nonsurgical periodontal therapy [92]. In addition, Qadri et al. [93] used two wavelengths, 636 and 830 nm, on patients with moderate gingival periodontitis and their results showed a decrease in the total amount of MMP-8 as compared to the placebo. From their study, they found PBM to ameliorate periodontal gingival inflammation. In a similar study, Qadri and colleagues [94] found that scale and root application together with Nd:YAG laser irradiation significantly reduced IL-1*β* and MMP-8 and therefore improved clinical signs linked with periodontal inflammation as compared to the treatment alone.

PBM is known for its stimulatory effects and promotes MMP activity and gene expression; hence maintaining a dynamic balance between the proteolytic activity and degradation could be a target for therapeutic advancement. However, its effect on various matrix proteins still needs to be further understood. Suggestions of further research in TIMPs and MMPs would be very important for the selection of these as indicators of early disease, in order to deduce standard protocols and improve clinical regimen [95].

## 6. Conclusion

This review covers the relationship between MMPs and their inhibitors in diabetic wound healing in relation to PBM and the ECM in both human and animal models,* in vivo* and* in vitro*. MMPs play a significant role in tissue remodelling. Given the fact that MMPs are known for degradation of the ECM and have been shown to be upregulated in most pathologies, they have been recognised as potential targets for new therapeutic measures. PBM can significantly restore the balance between the ECM and degradation enzymes. However, it is necessary to further elucidate the relationship between PBM, diabetic wound healing, and MMPs.
